# How should we interpret the data of eosinophilic esophagitis patients from an academic institution?

**DOI:** 10.1002/jgh3.12962

**Published:** 2023-08-29

**Authors:** Tadayuki Oshima

**Affiliations:** ^1^ Department of Gastroenterology Okazaki City Medical Association Public Health Center Okazaki Japan

Broadly speaking, findings obtained from a particular cohort of patients might not readily apply to the entire population, even when dealing with the same medical condition. In this regard, the data presented by Melgaard *et al*.^1^ in this issue of the journal seem well‐supported and credible. However, it is important to acknowledge that the applicability of their findings to a broader spectrum of individuals with eosinophilic esophagitis (EoE) within academic medical institutions is limited. This limitation stems from the fact that their study focused solely on patients referred to a single academic hospital. When seeking to identify characteristics of severe types of the disease, it is advisable to analyze data from tertiary care centers, as the severe forms of these diseases might not be adequately represented for analysis within the general population. Thus, while there may be differences between disease characteristics in the general population and those in tertiary care centers, the choice of study focus remains crucial. As studying patients referred to academic medical institutions is important, conducting multicenter studies involving participants from a wider range of geographic locations, socioeconomic backgrounds, and ethnicities enhances the applicability of study results to a broader population. In any case, understanding the attributes of these severe cases also becomes a pivotal aspect deserving attention, as they present potentially challenging or intricate clinical scenarios.

The global pooled incidence and prevalence of EoE were 5.3 cases per 100 000 inhabitant‐years (95% CI, 4.0–6.6) and 40.0 cases per 100 000 inhabitant‐years (95% CI, 31.1–49.0), respectively.^2^ While understanding the incidence and prevalence of EoE holds significance, it is essential to recognize that they distinctly vary in terms of lesions and diagnostic methodologies. Even within a single country, variations were observed in both the prevalence and incidence.^3^ These disparities could potentially stem from differences in diagnostic criteria, the utilization of diagnostic codes for disease classification, awareness levels regarding the condition, and the inclusion or exclusion of patients with proton pump inhibitor‐responsive esophageal eosinophilia (PPI‐REE).

EoE represents a chronic immune‐mediated inflammation of the esophagus, with type 2 inflammation induced by food and aeroallergens contributing to the dysregulation of esophageal epithelial barrier function.^4^ This chronic inflammation leads to tissue remodeling and fibrosis. However, the precise etiology of EoE remains unclear. The mean age at which EoE is diagnosed among adults is approximately 30 years.^3^ The temporal span between the onset of symptoms and the definitive diagnosis varies, spanning a spectrum of 3–8 years in the adult population. Notably, the study conducted by Melgaard *et al*.^1^ in this journal reported an average age of 40, and the significance of this difference remains unclear. Despite increased physician awareness leading to a rise in the incidence and prevalence of EoE, delays in diagnosing the condition still pose a significant challenge. The delay in diagnosing EoE is a major concern, particularly due to the correlation between the presence of esophageal strictures and the duration of untreated disease. Diagnostic delays are associated with the emergence of endoscopic findings such as rings and strictures, which may necessitate endoscopic treatment.^5^ The percentage of patients with stricture increased from 19% (diagnostic delay 2 years or less) to 52% (diagnostic delay 21 years or more) as demonstrated in a study conducted in the Netherlands. Additionally, each additional year of undiagnosed EoE was linked with a 9% increase in the probability of stricture development within that population.^6^ Several factors contribute to the risk of esophageal strictures in EoE, including delayed diagnosis, male gender, age at diagnosis, and a positive family history of the condition.^6^, ^7^ However, the incidence of stricture reported by Melgaard *et al*.^1^ in their study from Denmark appears to be lower.

The most commonly reported symptoms in adult EoE include dysphagia, food impaction, chest pain, and acid regurgitation. Symptom patterns have been found to vary by age and race. In terms of the impact of race on symptoms such as dysphagia and food impaction, both of these symptoms were more prevalent in Caucasians compared with African‐Americans and individuals of other races.^5^ Regarding differences based on sex, a report indicated that chest pain is more frequently reported by females, while dysphagia and food impaction are more common in males.^8^ However, it is important to note that the data supporting these differences were somewhat limited and remain subject to controversy. In any case, when patients present with these symptoms, it is crucial to consider EoE as a potential differential diagnosis.

The anticipated natural progression of EoE entails a transition from an inflammatory state to a fibrostenotic phenotype. However, no prospective study has been conducted due to ethical considerations. Furthermore, a study carried out in Sweden did not identify any increase in mortality rates among individuals with EoE.^9^ Consequently, the primary treatment strategy should focus on managing patients' symptoms and preventing the development of strictures. Advanced techniques for early detection and intervention of EoE, including the use of proton pump inhibitors and endoscopic dilation, have been recognized as effective strategies for preventing and managing EoE‐associated strictures.

While new biologics are being developed for eosinophilic gastrointestinal diseases (EGIDs), targeted therapies for EGIDs have proven to be relatively unsatisfactory compared with treatments for other autoimmune diseases. Currently, dupilumab is the only approved drug for treating EoE in the United States. These drugs have demonstrated histological improvements, but clinical trials have not shown sufficient symptom resolution.^10^, ^11^ Treatment responses have not been clearly defined, and normalizing mucosal eosinophil counts alone is not sufficient to achieve treatment goals.^12^ However, the normalization of the esophageal mucosal barrier might signal treatment success and the cure of EoE.^13^ Moreover, the rates of recurrence without treatment and the remission of EoE are still unclear. Consequently, in addition to raising awareness about EoE, the development of noninvasive diagnostic tests is crucial to prevent complications, recurrence, and assess treatment effectiveness.^14^
